# A rare complication of infantile hemangioma: Kasabach–Merritt phenomenon

**DOI:** 10.1093/jscr/rjae721

**Published:** 2024-11-25

**Authors:** Ricardo A Caravantes, José Manuel Toralla, Daniela Saenz

**Affiliations:** Department of Medical Research, Universidad Francisco Marroquín, 6ta calle final zona 10, Guatemala 01010, Guatemala; Department of Research, Universidad San Carlos de Guatemala, Guatemala 01010, Guatemala; Department of Medical Research, Universidad Francisco Marroquín, 6ta calle final zona 10, Guatemala 01010, Guatemala

**Keywords:** Kasabach–Merritt phenomenon, infantile hemangioma, vascular tumor, consumptive coagulopathy

## Abstract

Infantile hemangiomas are the most common type of vascular tumors, affecting ~5% of infants within the first weeks of life. In rare instances, these tumors can lead to Kasabach–Merritt phenomenon (KMP), a life-threatening consumptive coagulopathy characterized by thrombocytopenia, microangiopathic hemolytic anemia, and hypofibrinogenemia. In the present case, a 20-month-old patient is diagnosed with KMP. This case report highlights the challenges in diagnosis and management, reinforcing the importance of multidisciplinary approach.

## Introduction

Infantile hemangiomas (IH) typically present within the first weeks of life and are considered the most common type of vascular tumor in infancy [1]. They appear as raised, red or purple areas of skin and are more prevalent among girls, premature infants, and twins. While most IH follow a benign course and resolve spontaneously by the age of 5 to 10 years, some can become aggressive and develop complications such as Kasabach–Merritt phenomenon (KMP) [2]. KMP is characterized by severe thrombocytopenia and bleeding due to platelet trapping within the hemangioma, resulting in a consumptive coagulopathy that can be life-threatening [2]. The first-line treatment is propranolol, known for its high efficacy in reducing tumor size and associated complications [3]. However, alternative treatments, including corticosteroids, vincristine, and sirolimus, may be considered depending on the characteristics and response to initial therapy [3].

This case report describes a 20-month-old patient diagnosed with KMP, highlighting the clinical manifestations, diagnostic challenges, and treatment approach. The case emphasizes the importance of a multidisciplinary approach to manage such complex presentations effectively. Additionally, it underscores the need for early recognition of KMP to prevent severe complications and improve prognosis.

## Case report

A 20-month-old patient presented to the emergency department with a mass in the left maxillary region. The patient’s mother reported that a red-violet macule had developed in the left preauricular area and began to grow when the patient was 4 months old. At that time, laboratory tests revealed thrombocytopenia, leading to the initiation of propranolol therapy and platelet transfusions. Despite these treatments, the tumor continued to grow, eventually covering the periorbital region and causing edema in the ear pavilion.

**Figure 1:**
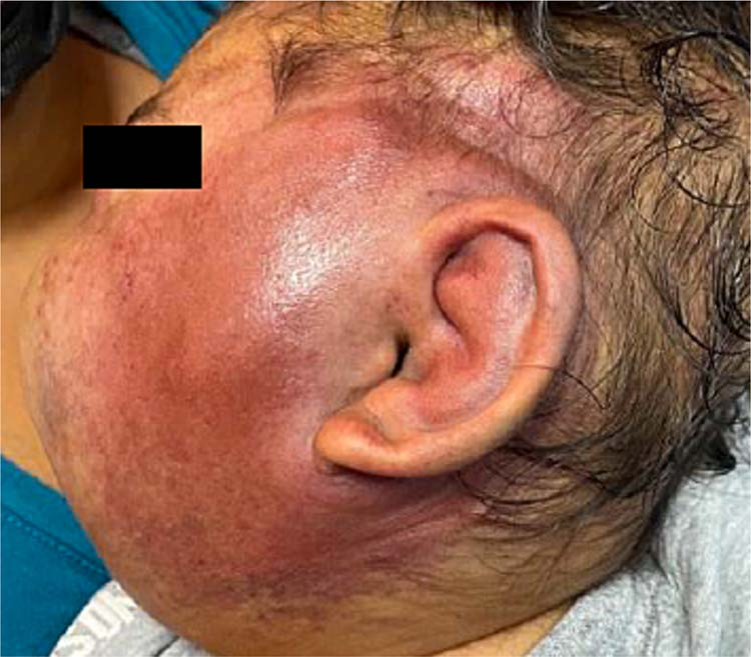
Hemangioma of ~15 × 20 cm, on left hemiface.

**Figure 2:**
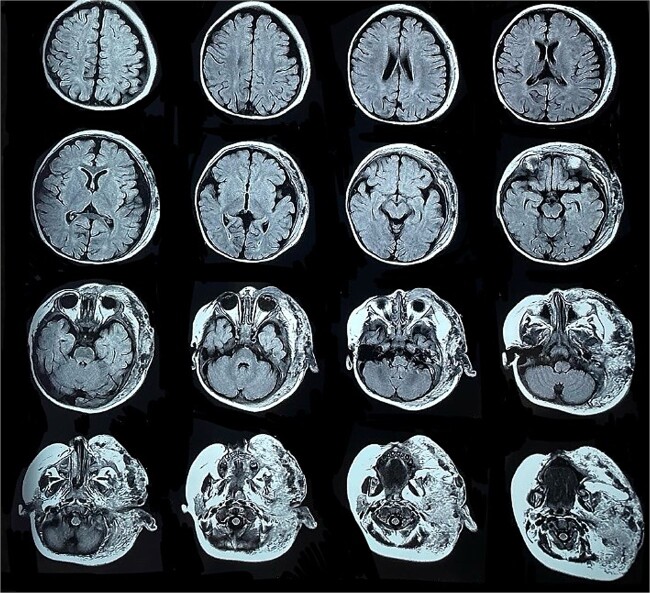
Angioresonance with evidence of a hemangioma on left hemiphase (cross-sectional).

**Figure 3:**
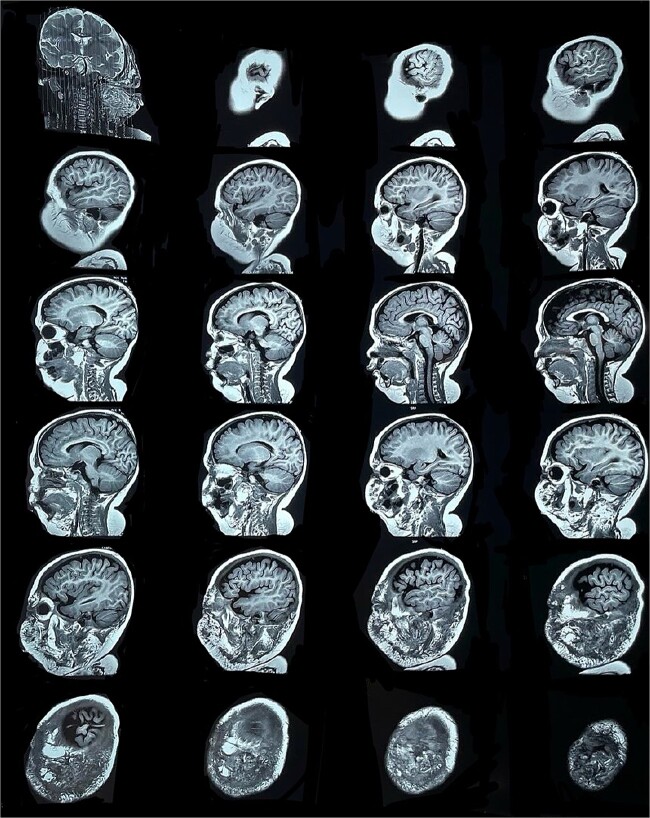
Angioresonance with evidence of a hemangioma on left hemiphase (coronal plane).

Six months later, the patient developed fever and severe edema that prevented the opening of the left eye, resulting in decreased overall tolerance. Laboratory results showed leukocytosis, severe thrombocytopenia (platelet count of 6000), and a positive C-reactive protein test. Physical examination revealed an irritable and lethargic patient with a 15 × 20 cm red mass with irregular borders (Fig. 1). An angioresonance was performed, revealing a hemangioma on the left hemiface that infiltrates muscle planes, the left parotid gland, and the auricular pavilion, ~10.7 × 6.6 × 13 cm in size, with possible vascularization from branches of the external carotid artery (Figs 2 and 3), leading to the diagnosis of KMP.

The patient underwent surgical intervention involving the ligation of the external carotid artery and resection of the hemangioma. The procedure resulted in a favorable outcome, and no recurrence has been reported.

## Discussion

The International Society for the Study of Vascular Anomalies (ISSVA) categorizes vascular anomalies (VA) into two primary groups: vascular tumors, defined by an increase in endothelial cell proliferation, and vascular malformations, defined by mesenchymal and angiogenic abnormalities [4, 5]. Vascular tumors can be benign, locally aggressive, borderline, or malignant.

IH are the most prevalent type of benign vascular tumor and the most common benign tumor in infancy, occurring in 5–10% of infants [1]. IH are more common in female infants, twins, caucasians, premature infants, infants with low birth weight, multiple pregnancies, older maternal age, preeclampsia, placental anomalies, and those with a family history of IH in a first-degree relative [1]. Typically, IH present within the first month of life and experience rapid proliferation with involution to adipose and fibrous tissue [6]. IH commonly develop in the epidermis, dermis, and subcutaneous fat, and are predominantly located in the head and neck (60%), trunk (25%), and extremities (15%) [7].

Diagnosing IH involves taking a thorough patient history and physical examination. The history should encompass the patient’s birth details, description of the initial lesion, the period of proliferation, and any complications experienced [1]. Imaging is recommended only for cases with challenging anatomical locations, diagnostic ambiguity, associated complications, or when PHACE or LUMBAR syndrome is suspected. IH appear as well-defined solid lesions on ultrasound (US) and magnetic resonance imaging; Doppler US can additionally provide insights into the lesion’s vascularity [1].

VA can be linked to hematological disorders like thrombocytopenia and/or consumptive coagulopathy, some of which result in KMP. This phenomenon has an incidence of 0.07/100000 and involves a rapidly growing hemangioma, thrombocytopenia, microangiopathic hemolytic anemia, and either acute or chronic consumptive coagulopathy, along with elevated D-dimer levels. It represents a form of consumptive coagulopathy caused by platelet trapping and aggregation [8].

The primary treatment for IH is propranolol, due to its efficacy and safety [9]. When it proves ineffective, alternative treatments such as topical corticosteroids, topical timolol, laser therapy, and surgery may be considered, depending on the hemangioma’s location, size, and behavior [3, 10]. Managing KMP is complex and necessitates a multidisciplinary approach. Treatment focuses on addressing the underlying hemangioma with systemic corticosteroids, vincristine, or other chemotherapeutic agents. Surgical intervention is typically considered when the tumor is resectable and does not present significant hemodynamic risks or involve critical local structures. Embolization might be considered if a single feeding vessel is present, but it is limited by the presence of multiple feeding vessels and the risk of local necrosis [2]. Supportive care, including platelet transfusions and coagulopathy management, is indicated primarily for patients who experience active bleeding or preparing for surgery [3, 11]. While profound thrombocytopenia is common in KMP, life-threatening hemorrhage is rare, and routine platelet transfusions are generally not recommended as they may exacerbate abnormal coagulation by becoming trapped in the tumor [11]. Symptomatic anemia should be managed with red blood cell transfusions, while cryoprecipitate may be used in situations of bleeding or preoperatively to address coagulopathy and mitigate bleeding risks [12].

Future studies should focus on the molecular and genetic profiling of hemangiomas that do not respond to standard treatments. Investigating alternative therapeutic strategies, including combination therapies or novel pharmacological agents, could provide better management options for such aggressive cases. Moreover, understanding the pathophysiology of KMP in the context of IH could lead to improved preventive measures and treatment protocols.

## Conclusion

IH are common benign vascular tumors in infants, characterized by their potential for rapid growth and subsequent involution. Despite their generally favorable prognosis, they can occasionally lead to severe complications such as KMP, which involves significant thrombocytopenia and consumptive coagulopathy. Understanding these pathologies is crucial for appropriate diagnosis and management.
